# Prediction Breast Molecular Typing of Invasive Ductal Carcinoma Based on Dynamic Contrast Enhancement Magnetic Resonance Imaging Radiomics Characteristics: A Feasibility Study

**DOI:** 10.3389/fonc.2022.799232

**Published:** 2022-05-19

**Authors:** Aqiao Xu, Xiufeng Chu, Shengjian Zhang, Jing Zheng, Dabao Shi, Shasha Lv, Feng Li, Xiaobo Weng

**Affiliations:** ^1^Department of Radiology, The Central Hospital Affiliated to Shaoxing University (Shaoxing Central Hospital), Shaoxing, China; ^2^Department of Surgical, The Central Hospital Affiliated to Shaoxing University (Shaoxing Central Hospital), Shaoxing, China; ^3^Department of Radiology, Fudan University Shanghai Cancer Center, Shanghai, China; ^4^Department of Research Collaboration, Research & Development Center (R&D), Beijing Deepwise & League of Doctor of Philosophy (PHD) Technology Co., Ltd, Beijing, China

**Keywords:** breast cancer, radiomics, omics analysis, LASSO regression algorithm, infiltration

## Abstract

**Objective:**

To investigate the feasibility of radiomics in predicting molecular subtype of breast invasive ductal carcinoma (IDC) based on dynamic contrast enhancement magnetic resonance imaging (DCE-MRI).

**Methods:**

A total of 303 cases with pathologically confirmed IDC from January 2018 to March 2021 were enrolled in this study, including 223 cases from Fudan University Shanghai Cancer Center (training/test set) and 80 cases from Shaoxing Central Hospital (validation set). All the cases were classified as HR+/Luminal, HER2-enriched, and TNBC according to immunohistochemistry. DCE-MRI original images were treated by semi-automated segmentation to initially extract original and wavelet-transformed radiomic features. The extended logistic regression with least absolute shrinkage and selection operator (LASSO) penalty was applied to identify the optimal radiomic features, which were then used to establish predictive models combined with significant clinical risk factors. Receiver operating characteristic curve (ROC), calibration curve, and decision curve analysis were adopted to evaluate the effectiveness and clinical benefit of the models established.

**Results:**

Of the 223 cases from Fudan University Shanghai Cancer Center, HR+/Luminal cancers were diagnosed in 116 cases (52.02%), HER2-enriched in 71 cases (31.84%), and TNBC in 36 cases (16.14%). Based on the training set, 788 radiomic features were extracted in total and 8 optimal features were further identified, including 2 first-order features, 1 gray-level run length matrix (GLRLM), 4 gray-level co-occurrence matrices (GLCM), and 1 3D shape feature. Three multi-class classification models were constructed by extended logistic regression: clinical model (age, menopause, tumor location, Ki-67, histological grade, and lymph node metastasis), radiomic model, and combined model. The macro-average areas under the ROC curve (macro-AUC) for the three models were 0.71, 0.81, and 0.84 in the training set, 0.73, 0.81, and 0.84 in the test set, and 0.76, 0.82, and 0.83 in the validation set, respectively.

**Conclusion:**

The DCE-MRI-based radiomic features are significant biomarkers for distinguishing molecular subtypes of breast cancer noninvasively. Notably, the classification performance could be improved with the fusion analysis of multi-modal features.

## 1 Introduction

According to the released data in 2020, breast cancer was the most common malignancy occurring in women worldwide and served as the main cause of cancer death (1). As one of the most common histological types of breast cancer, IDC approximately accounted for 80% of them. Patients who were diagnosed with the same pathological type and clinical stage of the disease may have distinct therapeutic outcomes due to tumor heterogeneity at the molecular level (2). Based on the expression of several specific molecular receptors, breast cancers are classified into three distinct molecular subtypes as follows: hormone receptor (HR)+/Luminal, HER2-enriched, and triple-negative breast cancer (TNBC). As the varied biological characteristics of these molecular subtypes, individuals generally respond differently to the same therapy (3). For example, patients with (HR)+/Luminal breast cancer subtype have the highest five-year survival rate and low recurrence risk, operation and endocrine therapy would be preferably suggested. For the patients with human epidermal HER2-enriched gene amplification, target treatment is strongly recommended to reduce the risk of recurrence. Given the strong invasion and the worst survival of TNBC subtype, neoadjuvant chemotherapy is recommended due to its relatively high sensitivity (4). In this context, early identification for the molecular subtypes could actively guide the targeted personalized therapy and prognostic prediction.

Clinically, immunohistochemistry is commonly used to determine the molecular type of breast cancer. However, it is invasive, and the molecular characteristics of the obtained tissue samples may fail to represent the overall tumor, and sometimes the molecular types of the specimens from the puncture and post-operation are inconsistent. Radiomics has been proven as an efficient noninvasive approach to correctly identify breast cancer molecular type. Dynamic contrast-enhanced magnetic resonance imaging (DCE-MRI) has been established as an imaging technique to present the morphologic and hemodynamic characteristics of tumors and is performed effectively to distinguish the tumor from the background parenchyma as the high-resolution of soft tissue (5, 6), thus it is commonly used for the feature extraction in radiomics (7). Prior studies have investigated radiomic signatures in the breast, Fusco et al. (8, 9) demonstrated that quantitative analysis of the morphology and texture features of breast lesions is feasible, a multiple classifier system can optimize the accuracy for breast lesion classification. Agner et al. (10) showed that good performances could be yielded using a probabilistic boosting tree classifier in conjunction with textural kinetic features for differential diagnosis between breast cancer and benign breast lesions. Some previous radiomics studies based on DCE-MRI (11–18) have already investigated the radiomic features of the breast, whereas the stability and reliability of models were affected by the difference in imaging schemes and devices. Furthermore, limited radiomic features or not complete subgroups of breast cancer in some previous studies resulted that the prediction performance of the provided models thus far are not the best. Therefore, this is still a lack of a comprehensive evaluation of MRI radiomic features for differentiating molecular types in patients with breast cancer.

This study aims to investigate the value of MRI radiomic features in distinguishing molecular types of breast cancer. To our knowledge, our study is the first attempt to extract radiomic features based on the original and wavelet-transferred DCE-MRI images through the 3D volumetric imaging technique, developing a nomogram combining radiomic characteristics and clinical pathological risk factors. Moreover, an external independent validation set was included to evaluate the stability of our models. We believe that our findings could provide valuable discriminative information of breast cancer molecular typings.

## 2 Materials and Methods

### 2.1 Patient Data

**Figure 1:**
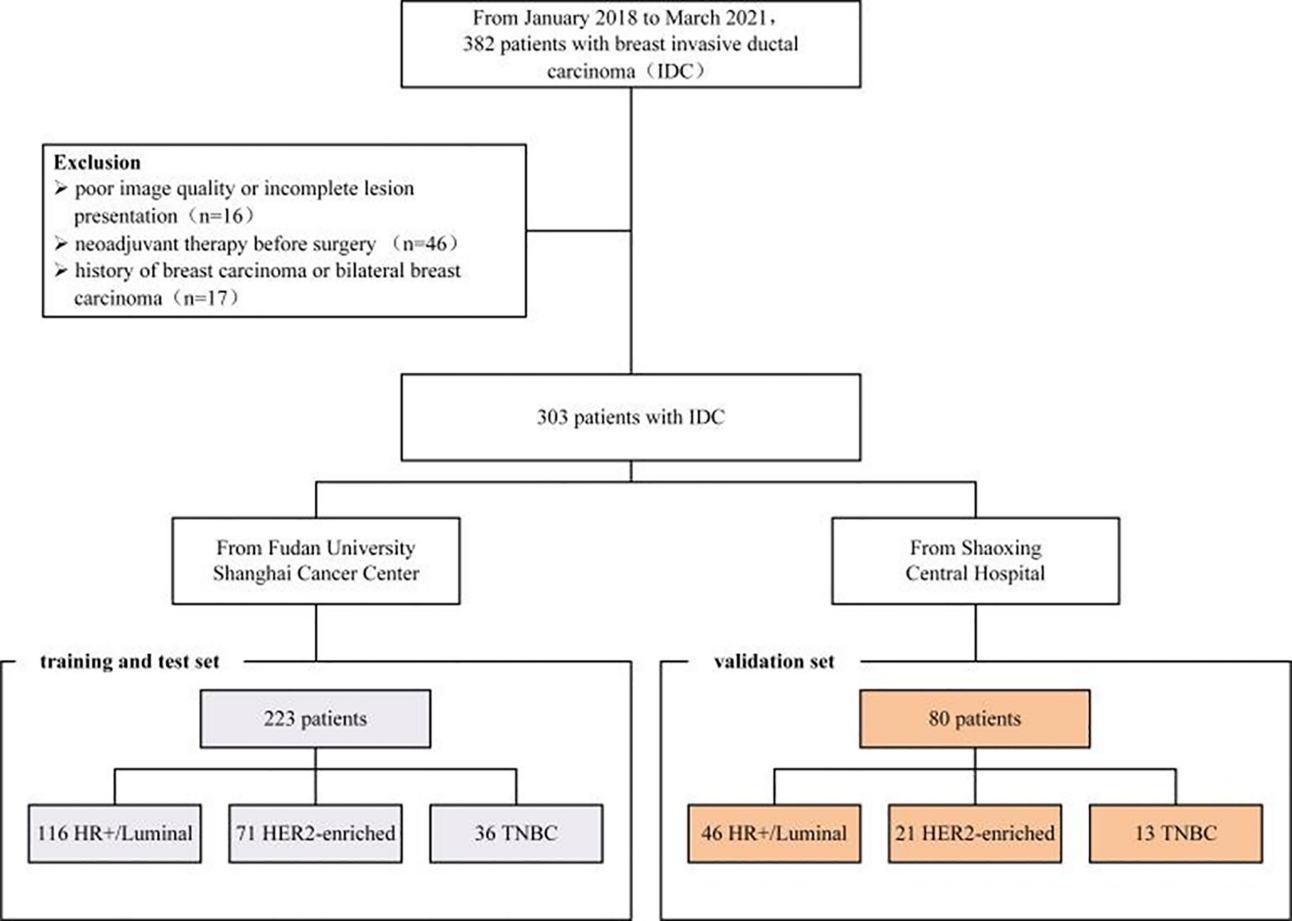
Patient workflow.

From January 2018 to March 2021, 382 patients diagnosed by clinical examination and confirmed by ultrasound in two hospitals were retrospectively included in this study. Patients were enrolled according to the following inclusion criteria: common breast invasive ductal carcinoma in pathology; complete breast MRI data, pathological and immunohistochemical data; and a long-term follow-up period. Exclusion criteria were pregnant or lactating females, or a plan to get pregnant within 6 months; prosthesis implantation; and a history of breast surgery that might affect imaging diagnosis. Of the finally included 302 patients, 223 cases (from Fudan University Shanghai Cancer Center) were randomly split into a training and internal test set with a ratio of 7:3, and 80 cases (from Shaoxing Central Hospital) were treated as an independent validation cohort. Demographic data from Electronic Medical Record Systems of both hospitals included age, menopause status, and tumor location. Pathological data included tumor pathological type and histological grade, status of estrogen receptor (ER) and progesterone receptor (PR), HER2, Ki-67, and lymph node metastasis. The study protocol was approved by the ethics committee of the Fudan University Shanghai Cancer Center and Shaoxing Central Hospital. The workflow of the patient selection process is given as **Figure 1**.

### 2.2 Imaging Examination

Fudan University Shanghai Cancer Center: Aurora Dedicated Breast MRI System and dedicated phase-array coil were used. The patients were asked to stay in the prone position to allow both mammary glands in the concave hole of phase-array coil with a natural overhanging effect. In the plain scan, cross-sectional T1-weighted images (T1WI) (TR 5 ms, TE 13 ms) and T2-weighted images (T2WI) with fat suppression (TR 6680 ms, TE 68 ms) were selected, with a layer thickness of 3 mm and a layer spacing of 1 mm. Phase I mask scan was performed before contrast enhancement scan. Gd-DTPA was used as the contrast agent at a dose of 0.2 mmol/kg and a flow rate of 2.0 mL/s. Contrast images at 5 phases were consecutively collected, with the scan time per phase as 120 s. In the contrast scan, cross-sectional T1WI with fat and water suppression (TR 5 ms, TE 29 ms) was selected, with a layer thickness of 1.1 mm and a layer spacing of 0, FOV 360 mm×360 mm, matrix 360×360×128. The number of scanned layers in a single phase was 160. Shaoxing Central Hospital: Philips Achieva 1.5T MR scanner (Holland) and dedicated breast coil were applied. In the plain scan, cross-sectional T1WI (TR 4.8 ms, TE 2.1 ms) and T2WI with fat suppression (TR 3400 ms, TE 90 ms) were selected, with a layer thickness of 3 mm and a layer spacing of 0.5 mm, matrix 512×512. Phase I mask scan was performed before contrast enhancement scan. Gd-DTPA was also used as the contrast agent at a dose of 0.2 mmol/kg and a flow rate of 2.0 mL/s. Contrast images at 6 phases were consecutively collected, with the scan time per phase was 90 s. In the contrast scan, cross-sectional T1WI with fat and water suppression (TR 5.0 ms, TE 2.2 ms) was selected, with a layer thickness of 1.0 mm and a layer spacing of 0.5 mm, FOV 320 mm×320 mm, matrix 336×336×128. The number of scanned layers in a single phase was 150.

### 2.3 Image Analysis

#### 2.3.1 Image Segmentation and Transfer

The breast DCE-MRI data were imported as DICOM file into the DeepWise scientific research platform v1.6 (http://keyan.deepwise.com/) to semi-automatically outline three-dimensional region of interests (3D ROIs) at the individual level and then revised manually by two radiologists of more than 10-years’ experience in breast imaging diagnosis. Disagreements were resolved by consensus-based discussion. The third sequence during the dynamic enhancement course was selected, the first series was acquired before intravenous injection, about 240 s for Aurora Dedicated Breast MRI System and about 180 s for Philips Achieva 1.5T MR scanner after injection of contrast medium. At this time point, malignant lesions show the general peak enhancement to present clear contrast with the surrounding normal breast parenchyma, which is conducive to more accurate ROI delineation and feature extraction. The chosen ROIs should conform to the following criteria: (1) Including cystic lesion, necrosis, and halo-sign; (2) invasion of surrounding structures: areas with connection to focus and have the same enhancement pattern with the focus; (3) reduction of volume effect for the upper and lower ends of focus: ROI <5 mm^2^ is waived. The coronal and sagittal planes could be further referenced to or getting calibration advice from superior physicians for decision making if there is any uncertainty. Finally, B-spline interpolation was carried out to standardize the image resolution into the same (1 mm × 1 mm × 1 mm) and followed by the gray-level discretization with fixed bin widths (25HU) as previous studies suggested.

#### 2.3.2 Radiomic Feature Extraction and Screening

To emphasize the imaging characteristics, three-dimensional wavelet decomposition was further applied at each level to obtain all possible combinations in high-pass or low-pass filters (LLH, LHL, LHH, HLL, HLH, HHL, HHH, LLL). For original and wavelet-transformed images, first-order, shape and texture features were extracted, respectively, which was implemented with open-source PyRadiomics library (https://github.com/Radiomics/pyradiomics). Subsequently, Z-score transformation was used to normalize the features distribution in the training set and the data in the other sets were then standardized by the same calculated parameters to avoid data-leakage. The implementation of feature extraction and standardization was in compliance with Imaging Biomarker Standardization Initiative (IBSI) (19).

Given the extracted high-throughput radiomic features, we initially applied feature selection in the training set to minimize the potential collinearity of variables and obtain the sparse feature matrix for modelling, which included Spearman’s rank correlation with a threshold of 0.9 and least absolute shrinkage and selection operator (LASSO) regression analyses, resulting in the most predictive covariates with non-zero coefficients.

#### 2.3.3 Model Establishment and Evaluation

Multi-class classification model was constructed using a transformed logistic regression. We transferred the multi-class cases into binary-class cases, hence there were three models: HR+/Luminal model (HR+/Luminal vs. rest), HER2-enriched model (HER2-enriched vs. rest), and TNBC model (TNBC vs. rest). We used the extended logistic regression method penalized by LASSO with 10-fold cross-validation to train the best performing classification models from the training set prior to external validation. To investigate the classification power of finally retained clinical and radiomic features, three multi-class models were built for classifying three primary molecular subtypes: clinical model, radiomic model, and combined model. Receiver operating characteristic (ROC) curves were used to evaluate the predictive discrimination in three molecular types with one-vs.-res (OvR) averaging strategy, which computes the average of the area under the curve (AUC) scores for each class against all other classes.

### 2.4 Pathological Analysis

Surgical specimens were obtained for pathological classification, histological grading, and immunohistochemical analysis. Molecular typing of breast cancer was performed according to the standard criteria proposed at the St. Gallen Conference (2, 20, 21): HR+/Luminal includes Luminal A and Luminal B, Luminal A for ER and/or PR+ (>1% staining) and HER2-; Luminal B for ER and/or PR+ (>1% staining) and HER2+; HER2-enriched for ER-, PR-, and HER2+, fluorescence *in situ* hybridization (FISH) was performed to assess gene amplification, and HER2 was considered positive if the ratio ≥ 2.0; and TNBC for ER-,PR-, and HER2-.

### 2.5 Statistical Methods

Statistical analysis was conducted on R statistical software v3.6.1 (http://www.Rproject.org). Student’s t test and Chi-square test were respectively used for continuous and categorical data with normal distribution, Mann-Whitney U test was applied for data with non-normal distribution. All tests were two-tailed, and a *p*-value threshold of 0.05 was considered statistically significant. The R package “glmnet” statistical software (R Foundation) was used to perform the modelling process of multi-class classification models. “PROC” R package was mainly used in the ROC curve analysis.

After the completion of feature selection for multi-class classification models, stepwise regression analysis based on Akaike Information Criterion (AIC) was devised to establish a nomogram for predicting molecular subtypes (HR+/Luminal and HER2-enriched)of breast cancer in the training set. The performance of the nomogram was evaluated by concordance index (C-index). Calibration curves of this nomogram were used to validate the agreement between prediction and observation in all data sets. Furthermore, we performed decision curve analysis (DCA) to visualize the net benefit for clinical decisions.

## 3 Results

### 3.1 Enhanced Imaging Data, Clinical Data, and Pathological Diagnosis Results

The detailed characteristics of patients are summarized in **Table 1**. In our study, all the cases were breast malignant tumors, 191 cases (85.7%) showed early enhancement with wash-in and rapid washout type curve or plateau type curve, and only 32 cases (14.3%) showed a slow increase followed by persistent enhancement curve. The mean age was 50.07 ± 10.48 years ranging from 16 to 86 years. Of the included 303 cases: HR+/Luminal (Luminal A, n=45; Luminal B, n=71) in 116 cases (52.02%), HER2-enriched in 71 cases (31.84%), and TNBC in 36 cases (16.14%). The HR+/Luminal breast cancer was the most prevalent subtype among them. There were 17 cases that were Stage I (7.62%), 114 cases were Stage II (51.12%), and 92 cases were Stage III (41.26%) in the histological grade assessment. There were no significant differences among the three subtypes in age (*P*=0.06) and histological grade (P=0.14). In contrast, the value of Ki-67 (*P*=0.01) and status of lymph node metastasis *(P*=0.03) were significantly different in the molecular subtypes of breast cancer.

**Table 1:** Clinical and histopathologic characteristics of patients grouped by molecular subtypes.

Characteristics	Total patients (N = 223, %)	Molecular subtypes			P value
		HR+/Luminal (N = 116, %)	HER2-enriched (N = 71, %)	TNBC (N = 36, %)	
Patient age	50.07 ± 10.48	51.62 ± 11.12	51.15 ± 8.98	45.91 ± 10.23	0.06
Histological grades:					
Stage I Stage II Stage III	17 (7.62) 114 (51.12) 92 (41.26)	12 (10.34) 74 (63.79) 30 (25.87)	2 (2.82) 18 (25.35) 51 (71.83)	3 (8.33) 22 (61.11) 11 (30.56)	0.14
Ki-67	37.42 ± 24.42	24.09 ± 16.48	45.91 ± 21.18	63.88 ± 23.17	0.01
Lymph node metastasis:					
Yes No	106 (47.5) 117 (52.5)	56 (47.8) 60 (52.2)	35 (49.3) 36 (50.7)	16 (44.4) 20 (55.6)	0.03
Menopause:					
No	83 (37.2)	41 (35.3)	25 (35.2)	17 (47.2)	0.399
Yes	140 (62.8)	75 (64.7)	46 (64.8)	19 (52.8)	
Position:					0.610
Central region	23 (10.3)	12 (10.3)	5 (7.04)	6 (16.7)	
Upper_right	63 (28.2)	33 (28.4)	19 (26.8)	11 (30.6)	
Lower_right	27 (12.1)	14 (12.1)	12 (16.9)	1 (2.78)	
Upper_left	82 (36.8)	42 (36.2)	26 (36.6)	14 (38.9)	
Lower_left	28 (12.6)	15 (12.9)	9 (12.7)	4 (11.1)	

### 3.2 Feature Selection and Optimal Omics Feature

**Figure 2:**
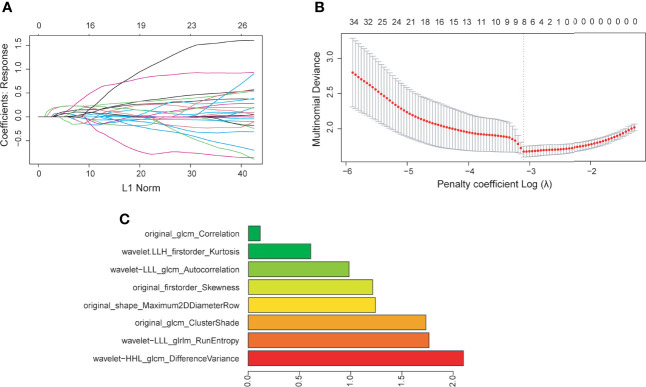
**(A)** Feature coefficients corresponding to the value of parameter λ. Each curve represents the change trajectory of each independent variable. **(B)** The most valuable features were screened out by tuning λ using LASSO regression with 10-fold cross-validation *via* minimum binomial deviation. The dotted vertical line represents the optimal log (λ) value. **(C)** The 8 selected radiomic features with the most discriminative value according to the best penalty parameter (λ).

Radiomic phenotyping of ROIs on the enhanced MRI images produced a total of 788 radiomic features from original and wavelet-transferred images, including first-order features (n=162), shape-order features (n=14), texture features from gray level co-occurrence matrix (GLCM, n=198), gray-level run length matrix (GLRLM) (n=144), gray-level size zone matrix (GLSZM) (n=144), and gray-level dependence matrix (GLDM) (n=126). Before feature selection, 48 (6%) radiomic features were excluded through stability analysis (ICC≤ 0.85). There were 148 radiomic features and 6 clinical features selected with the |correlation coefficient| ≤ 0.9. **Figure 2** shows the selection process where the subset size of non-zero features tuned by the parameter λ is based on the minimum criteria. The optimal λ (log (λ) = −3.331) resulted in 8 radiomic features with non-zero coefficients (**Figure 2C**). We further verified that there was no statistically significant difference in those features between the training set and test set (**Table 2**).

**Table 2:** Analysis of the selected texture features in the training and test sets.

Characteristics	Training set	Test set	P value
original_glcm_ClusterShade	1551.28 ± 367.89	1426.08 ± 355.01	0.09
original_shape_Maximum2DDiameterRow	32.32 ± 16.40	29.56 ± 12.31	0.23
original_firstorder_Skewness	-0.22 ± 0.51	-9.24 ± 0.45	0.13
wavelet.LHL_firstorder_Kurtosis	3.83 ± 1.42	3.87 ± 1.28	0.26
original_glcm_Correlation	0.27 ± 0.16	0.23 ± 013	0.15
wavelet-LLL_glcm_Autocorrelation	14619.04 ± 7738.10	15728.27 ± 8115.49	0.08
wavelet-LLL_glrlm_RunEntropy	6.74+0.69	6.67 ± 0.63	0.35
wavelet-HHL_glcm_DifferenceVariance	142.67 ± 115.07	175.65 ± 124.28	0.16

### 3.3 Model Construction and Validation

**Figure 3:**
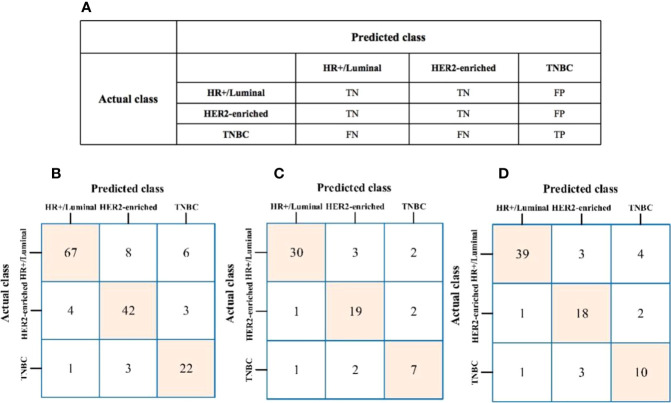
**(A)** The prediction results of confusion matrix when TNBC was labeled as the target. TP, true positive; TN, true negative; FP, false positive; FN, false negative. **(B–D)** Confusion matrix of the combined model to the training, test, and external validation sets, respectively.

**Figure 4:**
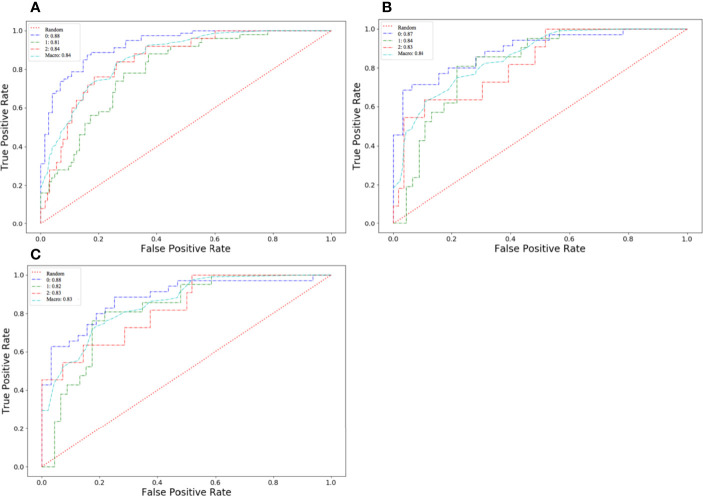
Receiver operating characteristic (ROC) curves of the combined model in distinguishing molecular subtypes of breast cancer. **(A)** The training set. **(B)** The test set. **(C)** The external validation set.

The algorithm of extended logistic regression penalized by LASSO finally determined 8 optimal radiomic features (**Table 2**) and 4 clinical features (age, tumor location, histological grade, Ki-67, and lymph node metastasis). Three multi-class classification models (clinical model, radiomic model, and combined model) were constructed considering not only single-modal features but also the fusion of multi-modal features. The confusion matrix of the combined model shown in **Figure 3** demonstrates that the proposed multi-class model performs well on most one-vs.-res (OvR) results. For predicting molecular subtype, the model performance for classifying HR+/Luminal vs. non-HR+/Luminal, HER2-enriched vs. non-HER2-enriched, and TNBC vs. non-TNBC in the three data sets is shown in **Table 3**. The ROC analyses for the combined model in distinguishing molecular subtypes of breast cancer are shown in **Figure 4**. For the combined model, the value of macro-AUC was 0.84 (95%CI: 0.80-0.90) in the training set and 0.84 (95%CI: 0.77-0.86) in the test set. For the radiomic model, the value of macro-AUC was 0.81 (95%CI: 0.78-0.87) in the training set and 0.81 (95%CI: 0.75-0.87) in the test set. For the clinical model, the value of macro-AUC was 0.71 (95%CI: 0.67-0.79) in the training set and 0.73 (95%CI: 0.68-0.79) in the test set. In the external validation set, the combined model yielded the highest value of macro-AUC (0.83, 95% CI: 0.77-0.89) (**Figure 4**).

**Table 3:** ROC values of three models for distinguishing molecular subtypes of breast cancer.

	Molecular subtypes	Training set (n = 156)		Test set (n = 67)		Validation set (n = 80)	
		AUC	95%CI	AUC	95%CI	AUC	95%CI
Clinical model	HR+/Luminal HER2-enriched TNBC macro-averaging	0.75 0.71 0.69 0.71	0.67-0.79 0.66-0.78 0.63-0.74 0.67-0.79	0.77 0.74 0.70 0.73	0.68-0.79 0.70-0.78 0.64-0.77 0.68-0.79	0.76 0.77 0.72 0.76	0.71-0.79 0.73-0.84 0.68-0.76 0.72-0.82
Radiomic model	HR+/Luminal HER2-enriched TNBC macro-averaging	0.81 0.84 0.83 0.81	0.78-0.87 0.80-0.88 0.76-0.86 0.78-0.87	0.81 0.82 0.80 0.81	0.75-0.87 0.77-0.84 0.79-0.86 0.75-0.87	0.79 0.85 0.78 0.82	0.72-0.86 0.80-0.89 0.74-0.82 0.76-0.88
Combined model	HR+/Luminal HER2-enriched TNBC	0.84 0.88 0.81	0.80-0.90 0.85-0.92 0.77-0.98	0.83 0.87 0.84	0.77-0.86 0.82-0.90 0.80-0.89	0.83 0.88 0.82	0.79-0.86 0.84-0.89 0.78-0.86
	Macro-averaging	0.84	0.80-0.90	0.84	0.77-0.86	0.83	0.77-0.89

### 3.4 Nomogram Establishment

**Figure 5:**
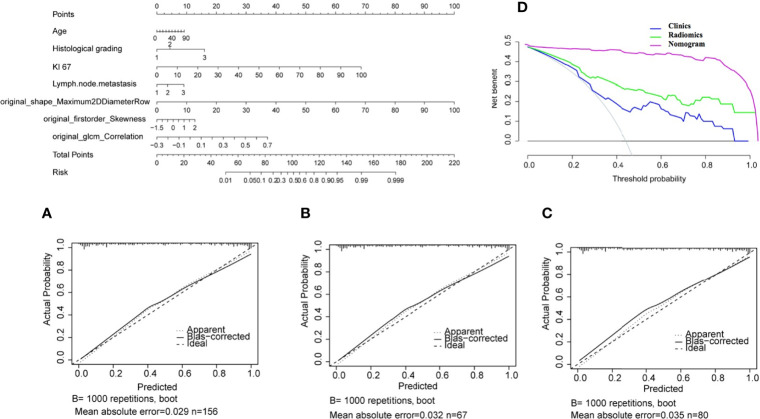
Nomogram for classifying HR+/Luminal and HER2-enriched molecular subtypes of breast cancer. **(A–C)** Calibration curves of the nomogram in the training, test, and external validation sets, respectively. **(D)** Decision curve analysis of the nomogram.

The nomogram for the classification model of HR+/luminal and HER2-enriched is shown in **Figure 5**, in which original_shape_Maximun2DDiameterRow has the most discriminative power, and the value of C-index was 0.84 in the external dependent validation set. The calibration curves of the combined nomogram showed good calibration performances in the training set, test set, and external validation set, the high agreements between ideal curves and calibration curves were observed. The DCA curve revealed a more extensive range of cutoff probabilities shown by the nomogram, the threshold probabilities of the model had excellent net benefits and enhanced performance for classifying the two molecular subtypes with combined nomogram.

## 4 Discussion

Radiomics is a rapid developing field of medical study that quantitates the microstructure and biological information of tumor tissue for exploring the intra-tumoral heterogeneity and tumor characterization in a convenient and non-invasive way (22). To date, some studies have already investigated the discrimination between benign and malignant breast tumors (23, 24), lymph node metastasis (25–27), tumor response prediction of neoadjuvant chemotherapy (28, 29), and survival analysis (30, 31). Our study found that radiomics showed favorable predictive performance on molecular subtype based on the DCE-MRI images. In the present study, we identified 8 radiomic features as significant in the radiomics model and 4 clinical features in the clinical model. The combined model with the fusion of clinical and radiomic features was proven to have the optimal performance in distinguishing molecular subtype of breast cancer, with the value of sensitivity, specificity, and macro-AUC were 0.832, 0.781, and 0.830, respectively. Furthermore, based on the optimal radiomic features and clinical risk factors (patient age, pathological grade, Ki-67, and lymph node metastasis), a clinical predictive nomogram for Her2+/Luminal molecular subtypes was constructed. DCA, a method available to obtain net benefit based on threshold probability, revealed the superiority of the nomogram in the classification of molecular subtype of breast cancer. To validate the stability and reliability of all models, further testing was applied in the internal test set and an independent external validation set, the nearly similar values of macro-AUC indicating the excellent robustness and generalization, meaning good practical value for molecular subtype classification in coming breast cancer cases.

Previous studies (32–34) have suggested that MRI-based radiomic features are definitely correlated with the molecular subtypes of breast cancer. Wu et al. (35) selected the largest tumor from the fourth sequence during the dynamic enhancement course and obtained the accuracy of 0.786, 0.733, and 0.941 in distinguishing between Luminal A and non-Luminal A, Luminal B and non-Luminal B, and TNBC and non-TNBC. However, a small sample size (79 cases) and no additional independent validation set resulted that the reproducibility and reliability of the models were needed to be further verified. Li et al. (7) achieved good results in distinguishing between ER+ and ER- based on a radiomic signature (AUC=0.89), although only leave-one-out cross validation (LOOCV) was used because of the limited cases. Rossana et al. (36) investigated three advanced machine learning algorithms, including support vector machine, random forest, and Naive Bayes classifier, and successfully identified the molecular prognostic markers (AUC: 86-93%). The results from the previous studies are not completely consistent, probably influenced by the difference in selected phase/level in contrast scan, or the method for molecular typing. In contrast, a few studies provided different views. Grimm et al. (11) assessed the value of imaging features in predicting molecular subtypes of breast cancer from three aspects, including morphology, radiomics, and dynamic enhancement, by a semi-automated segmentation approach (i.e., fuzzy C-means clustering). They found that radiomic features were inferior to the other two types of features. The discrepancy between the views might be caused by the changes in the scanner and the pulse sequence applied in the studies. Notably, it has been proven that the matrix size is crucial in feature calculation as its relationship with spatial resolution. The model in our study provided high accuracy, which is consistent with the study of Leithner et al. (33), which could be interpreted as follows: First, in the most obvious enhancing phase, the heterogeneity and invasiveness of the tumor will be reflected obviously (32), and the much clearer boundary of focus will minimize errors occurring in focal delineation. Second, the semi-automated segmentation approach for the extraction of breast DBT and 3D ROI on MRI original images is more reliable compared to the other 2D or the maximum level analyses in the same research field. Third, the additional exploration of wavelet-based features revealed some more specific image characteristics of overall lesions.

In this study, an extended logistic regression with LASSO penalty was applied to obtain 8 optimal radiomic features from the total 788 candidate radiomic features. The 8 features include morphological, first-order, GLCM and GLRLM characteristics, which are predominantly related to tumor heterogeneity. Shape_Maximum 2D Diameter Row depicts the tumor size and morphology, which was proved significantly correlated with the molecular type of breast cancer, indicating the molecular subtype could be influenced by tumor size. Consistent with the previous report (37), the morphology and size of lesions varied with the expression of different hormone receptors, and hormone receptor-negative plus HER2-positive or TNBC breast cancers tend to have larger lesions than hormone HR+/Luminal cancers. We also found that the low kurtosis and skewness appeared in HR+/Luminal cases, which are highly important in the radiomic model. Compelling evidence provided by Fan et al. (14), who constructed a predictive model for four molecular subtypes of breast cancer based on DCE-MRI radiomic, dynamic, and 2 clinical features, revealed the heterogeneity-related low kurtosis and skewness in Luminal A cases and highlighted the potential of skewness as a predictor for molecular subtype classification of breast cancer. It is reported that higher kurtosis and skewness values are associated with treatment failure (38), while lower values indicate good responses to treatment. This is supported by the fact that HR+/Luminal breast cancers have favorable clinical outcomes. Correlation, Autocorrelation, DifferenceVariancewavelet and glrlm_RunEntropy are second-order or high-order features based on original and wavelet transforms. They reflect the roughness of texture and the consistency between tumor texture images, conducive to better predicting intra-tumor heterogeneity and subtle differences in gray level texture feature (13). Moreover, they are regarded to be of vital significance in texture analysis in the field of medical imaging.

There are still some limitations in this study. For instance, only the phase with the most obvious dynamic enhancement was selected for analysis. The further requirement of T1WI, T2WI, and DWI images, which are essential in breast cancer analysis, may provide a more comprehensive information of the lesions. In future research, a complete sequence will be involved to further investigate the value of multi-parameter radiomic features in predicting molecular subtype of breast cancer. Another limitation is that the TNBC showed an unbalanced distribution in all breast cancers, though it reflected the general distribution of breast cancer molecular subtypes in the patient population. Hence, we adopted the cross-validation method to ensure the stability of results in a different split training cohort.

To sum up, the radiomics signature based on DCE-MRI has good clinical application value in predicting molecular subtype of breast cancer, and it may help clinicians make beneficial treatment decisions before surgery.

## Data Availability Statement

The raw data supporting the conclusions of this article will be made available by the authors, without undue reservation.

## Ethics Statement

The study protocol was approved by the ethics committee of the Fudan University Shanghai Cancer Center and Shaoxing Central Hospital. Written informed consent to participate in this study was provided by the participants’ legal guardian/next of kin.

## Author Contributions

AX and XW conceived and designed this study. XC carried out to collect the clinical data. JZ collected pathological data. AX and XC drafted the manuscript. FL performed the statistical analysis. DS performed image processing. SZ and SL put forward many opinions on the manuscript. All authors contributed to the article and approved the submitted version.

## Funding

This work was supported by the Medical and Health Research Project of Zhejiang Province (Grant No. 2021KY1161, 2022KY1316); Zhejiang Province Chinese Medicine Science Research Fund Project (Grant No. 2021ZA138); and institution from Key Laboratory of Functional Molecular Imaging of Tumor and Interventional Diagnosis and Treatment of Shaoxing City.

## Conflict of Interest

Author FL was employed by the Beijing Deepwise & League of PHD Technology Company.

The remaining authors declare that the research was conducted in the absence of any commercial or financial relationships that could be construed as a potential conflict of interest.

## Publisher’s Note

All claims expressed in this article are solely those of the authors and do not necessarily represent those of their affiliated organizations, or those of the publisher, the editors and the reviewers. Any product that may be evaluated in this article, or claim that may be made by its manufacturer, is not guaranteed or endorsed by the publisher.
