# Supplementary material for: Prediction Breast Molecular Typing of Invasive Ductal Carcinoma Based on Dynamic Contrast Enhancement Magnetic Resonance Imaging Radiomics Characteristics: A Feasibility Study
Source: Front Oncol. 2022 May 19;12:799232. doi: 10.3389/fonc.2022.799232 (PMC9160981; doi:10.3389/fonc.2022.799232)
Supplement: Supplementary file 1 [file Table_1.docx]

Supplementary Material

**Supplementary Table S1.** Predictive effectiveness of each model in the training and test sets

|  | Training set | | Test sets | |
| --- | --- | --- | --- | --- |
|  | Sensitivity | Specificity | Sensitivity | Specificity |
| Clinical Model | 64.16% | 71.67% | 60.14% | 70.10% |
| Radiomic Model | 74.11% | 77.20% | 80.55% | 81.98% |
| Combined Model | 82.37% | 80.26% | 83.21% | 78.05% |
